# Development of Hematopoietic Stem Cell Based Gene Therapy for HIV-1 Infection: Considerations for Proof of Concept Studies and Translation to Standard Medical Practice

**DOI:** 10.3390/v5112898

**Published:** 2013-11-22

**Authors:** David L. DiGiusto, Rodica Stan, Amrita Krishnan, Haitang Li, John J. Rossi, John A. Zaia

**Affiliations:** 1 Department of Virology, Beckman Research Institute of the City of Hope, 1500 East Duarte Road, Duarte, CA 91010, USA; E-Mails: rstan@coh.org (R.S.); jzaia@coh.org (J.A.Z.); 2 Department of Hematology and Hematopoietic Cell Transplantation, City of Hope, 1500 East Duarte Road, Duarte, CA 91010, USA; E-Mail: akrishnan@coh.org; 3 Department of Molecular and Cellular Biology, Beckman Research Institute of the City of Hope, 1500 East Duarte Road, Duarte, CA 91010, USA; E-Mails: hli@coh.org (H.L.); jrossi@coh.org (J.J.R.)

**Keywords:** HIV-1, stem cells, gene therapy, non-ablative conditioning, transplantation

## Abstract

Over the past 15 years we have been investigating an alternative approach to treating HIV-1/AIDS, based on the creation of a disease-resistant immune system through transplantation of autologous, gene-modified (HIV-1-resistant) hematopoietic stem and progenitor cells (GM-HSPC). We propose that the expression of selected RNA-based HIV-1 inhibitors in the CD4+ cells derived from GM-HSPC will protect them from HIV-1 infection and results in a sufficient immune repertoire to control HIV-1 viremia resulting in a functional cure for HIV-1/AIDS. Additionally, it is possible that the subset of protected T cells will also be able to facilitate the immune-based elimination of latently infected cells if they can be activated to express viral antigens. Thus, a single dose of disease resistant GM-HSPC could provide an effective treatment for HIV-1+ patients who require (or desire) an alternative to lifelong antiretroviral chemotherapy. We describe herein the results from several pilot clinical studies in HIV-1 patients and our strategies to develop second generation vectors and clinical strategies for HIV-1+ patients with malignancy who require ablative chemotherapy as part of treatment and others without malignancy. The important issues related to stem cell source, patient selection, conditioning regimen and post-infusion correlative studies become increasingly complex and are discussed herein.

## 1. Introduction

Acquired immunodeficiency syndrome (AIDS) is the disease caused by infection with the human immunodeficiency virus type 1 (HIV-1). According to the World Health Organization (WHO), there were 34 million people living with AIDS, with 2.7 million new cases and 1.8 million deaths worldwide in the year 2010 [1]. Contemporary therapeutic intervention is aimed at controlling viral replication and preserving immune function through the use of a cocktail of antiretroviral drugs known as combination antiretroviral therapy (cART). However, patients with well controlled viremia on cART (<50 infectious units/mm^3^ of blood) are known to harbor a latent viral reservoir in resting CD4+ T cells [2,3,4,5]. When cART is interrupted, latently infected cells produce infectious virus that can be rapidly followed by a loss of peripheral blood CD4+ T cells and progression towards immunodeficiency. Additionally, the prolonged use of cART is associated with other clinical sequelae, including neural, renal, hepatic and cardiovascular toxicity, diabetes, lipodystrophy and other metabolic abnormalities [6,7,8,9,10]. Moreover, the widespread use of cART and a lack of patient compliance with drug treatment schedules have resulted in the development of viral variants (escape mutants) that are drug resistant. Together, these limitations of cART emphasize the need for a more comprehensive approach to HIV-1 therapy.

*Allogeneic Stem Cell Protection from HIV-1.* One method for achieving an HIV-1 resistant immune system is to transplant patients with allogeneic hematopoietic stem and progenitor cells (HSPC) that are naturally resistant to HIV-1 infection. Individuals with a homozygous (32 base pair) deletion in the coding region of the CCR5 gene (CCR5^∆32/∆32^), the co-receptor for (R5) tropic HIV-1 viral entry produce CD4+ progeny that are resistant to R5 tropic HIV-1 infection [11]. Using this approach, Hutter *et al*. described a patient with acute myeloid leukemia, who was cured of AIDS following a bone marrow transplant from an HLA-matched, unrelated donor (URD) with a CCR5^∆32/∆32^ genotype. It is not clear whether it was the CCR5 mutational status alone or some additional elements of the transplant procedure, e.g., use of anti-T cell therapy for graft *vs.* host disease (GVHD) prophylaxis or the GVHD, that contributed to this cure. Nevertheless, there is general consensus that the treatment conferred long-term control of HIV-1 replication as the patient has been off cART for over 4 years without detectable HIV-1 [12,13]. Of interest, subsequent observations of apparent HIV-1 control following allogeneic HSPC transplantation from URD with wild-type CCR5 genotype, have suggested that the allogeneic effect contributes to the cure, analogous to the graft *vs*. leukemia effects in these same patients [14]. From a practical standpoint, the difficulty in identifying HLA-matched, CCR5^∆32/∆32^ donors for transplantation, the significant risks associated with GVHD, the co-morbidities of myeloablative allogeneic transplant, and the cost preclude the general application of this approach. Moreover, based on a review of anecdotal use of allogeneic HSPC transplantation in AIDS patients [15], there are other concerns with this strategy such as (a) the need for chronic immunosuppression for GVHD-prophylaxis when the AIDS patient is already immunodeficient; (b) the pharmacologic interactions of cART during conditioning therapy or with the GVHD immunosuppressant medications; and (c) the potential that late-stage HIV-1 infection will enhance the rate of transplant-related complications, including graft failure or inability to establish immune reconstitution. We hypothesize that a more practical approach to control of viremia and restoration of healthy levels of immune function in HIV-1+ patients may be achieved through transplantation of gene modified (HIV-1 resistant) autologous HSPC. Several groups (including our own) have “engineered” resistance to HIV-1 through inhibiting viral entry, transcription, transport of viral RNA and other HIV-1 specific mechanisms (reviewed in [16]).

*Autologous Stem Cell Protection from HIV-1.* Proof of concept for the creation of an HIV-1-resistant immune system has been repeatedly demonstrated using several different “humanized” mouse models and cord blood or fetal liver HSPC [17,18,19,20,21], but only a few clinical studies have been conducted. In early clinical studies performed in pediatric patients, autologous HSPC were genetically modified using a retroviral vector that expressed either a RRE decoy [22] or transdominant Rev (RevM10) [23] and then transplanted without myelosuppressive conditioning. The safety of the procedure was demonstrated in the first (RRE Decoy) study, but the level of engraftment of gene-marked cells in the peripheral blood was transient, lasting only a few months in most patients and well below the level of quantification (10^−4^–10^−5^ copies/cell). In the second (RevM10) study, gene expression was also transient and too low to quantify after the first three months. Gene marking in two pediatric patients returned to detectable levels following suspension of anti-retroviral therapy and an episode of acute viremia, suggesting that viral recrudescence can lead to enrichment of HIV-1-resistant cells. In a similar series of studies in adult patients, HSPC were transduced with a retroviral vector encoding a ribozyme directed against the HIV-1 *vpr* and *tat* sequences and also transplanted without prior marrow conditioning [24]. The first 10 patients were all successfully engrafted and had detectable gene marking in the peripheral blood for up to 3 years, although the levels were again in the 10^−4^–10^−5^ copies/cell range and (in general) not quantifiable. In a follow-up (Phase II) trial, 74 patients received either anti-HIV-1 ribozyme or placebo gene therapy, again without any myelosuppressive conditioning [25]. While the primary endpoints were not reached in this study (control of viral load 47–48 weeks after transplant), a decrease in the viral load and transient improvements in CD4 count (as assessed by total weighted area under the curve) were observed in the ribozyme treated but not the control group upon analytical treatment interruption (ATI) of cART. These two studies demonstrate that HSPC (and their progeny) can be isolated, genetically-modified and used to engraft patients in the absence of myelosuppressive conditioning. However, the low levels of engraftment preclude realization of sufficient clinical benefit to warrant long-term suspension of cART.

## 2. Results and Discussion

### 2.1. Initial Trial Using Lentiviral Vectors and Ablative Conditioning

**Figure 1:**
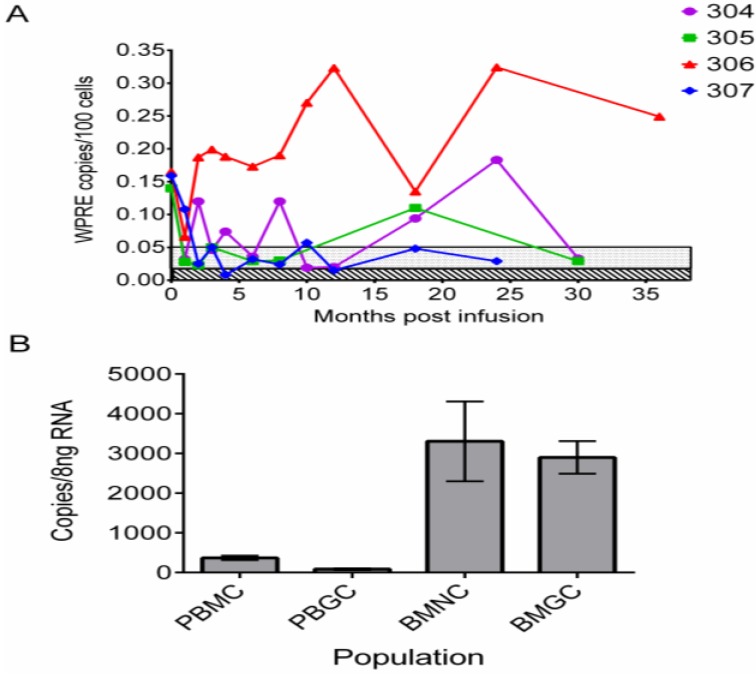
Sustained engraftment and expression of anti-HIV genes in the peripheral blood of patients for up to three years after transplantation. (**A**) Level of gene marking expressed as number of copies of vector (WPRE) per 100 blood cells analyzed over time. Unique patient identifiers are listed in the upper right hand corner of graph. Limits of quantification (stippled) and limits of detection (diagonal lines) values were determined for each amplification reaction and typically were in the range of 0.05% (500 cells/million) and 0.01% (100 cells/million), respectively. (**B**) Expression of shRNA against *tat/rev* sequences in the blood and marrow of UPN0306 at 36 months. Results expressed as copies of shRNA per 8 ng of total RNA. PBMC—peripheral blood mononuclear cells, PBGC—peripheral blood granulocytic cells, BMMC—bone marrow mononuclear cells, BMGC—bone marrow granulocytic cells. Bar height in B is mean of triplicates ± SEM.

We previously reported a “first in human” clinical trial to assess the safety and feasibility of lentivirus-transduced autologous stem cell gene therapy for HIV-1 in patients undergoing autologous stem cell transplantation for AIDS related lymphoma [26]. We reasoned that (a) transplanting patients who require high-dose (marrow ablative) chemotherapy as part of their lymphoma therapy would result in a higher level of engraftment of the gene-modified cells; and (b) the risk:benefit ratio in these patients was favorable for first in human studies. CD34+ HSPC from 5 patients were genetically modified using a lentiviral vector encoding a short hairpin RNA (shRNA) targeting HIV-1 *tat* and *rev* gene sequences, a nucleolar localizing TAR decoy and a CCR5-specific hammerhead ribozyme [27]. We were successful in preparing GM-HSPC products for infusion in four of five patients and all patients transplanted had detectable gene marking and expression at one or more time points for up to two years. In one patient (UPN0306), we have now observed long-term (>3 years) quantifiable gene marking and expression of anti-HIV-1 siRNA in peripheral blood cells (Figure 1).

**Figure 2:**
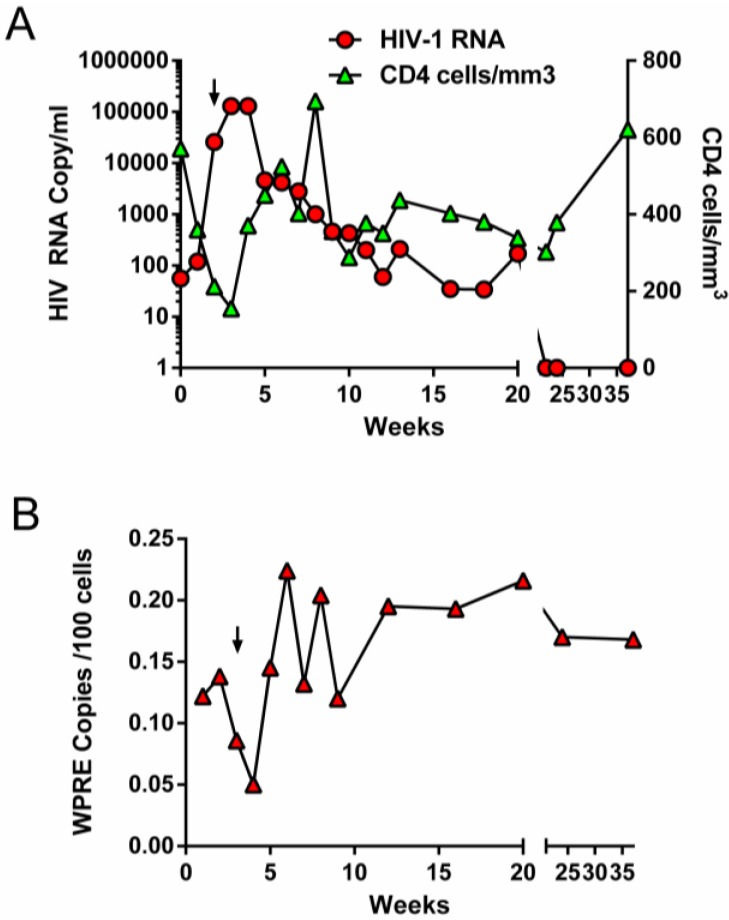
Monitoring of viral loads, CD4 counts and gene marking during structured treatment interruption. (**A**) HIV copies/mL of patient plasma (red circles, left Y axis) and CD4 counts/mm^3^ (green triangles, right Y axis) during treatment interruption. (**B**) Gene marking expressed as copies of vector (WPRE) per 100 blood cells analyzed over time. Arrow indicates time of resumption of cART.

Unfortunately, the initial gene marking level of the more primitive hematopoietic stem cells was low (~1% as estimated by PCR analysis of cells from 5-week stromal cell co-cultures). Furthermore, the GM-HSPC were infused with a 50–100 fold excess of unmanipulated HSPC (as a safety measure in this first in human trial), which resulted in a low frequency of gene-modified progeny cells (0.1%–0.3%) in the peripheral blood and marrow of these patients. However, the level of gene marking was significantly higher (10^−3^–10^−4^) than the previously described studies using retroviral vectors with no marrow conditioning and demonstrates the utility of lentiviral vectors and marrow ablation in enhancing the levels of gene-modified cells obtained *in vivo*. Patient UPN0306 also underwent ATI to assess the control of viremia and potential for preserving CD4 cell counts in the absence of cART. We observed a rapid spike of virus four days after suspension of cART followed by a sharp decline in CD4 levels to below 200 cells/mm^3^ (Figure 2).

The patient was placed back on cART 21 days into the treatment interruption and CD4 levels recovered to >600/mm^3^ while viral load dropped to below the level of detection. Similar to the RevM10 study described above, we also observed a transient increase in the level of gene modified cells in the peripheral blood from ~0.125% to ~0.225% during this period, which did not reach statistical significance. Analysis of vector integration 5 weeks after initiation of ATI (2 weeks after resumption of cART) revealed that although 10 independent integration sites were identified, two clones accounted for virtually all of the gene marking seen in this patient at this time (Table 1). The first clone had vector integrated in an intronic region of the uncharacterized protein gene (KIAA1683) and the second clone, equally represented, had vector integrated in an intron of a C2H2 type zinc finger protein gene (ZNF559). The integration patterns of all identified sites are consistent with previous findings for integration of lentiviral vectors in largely non-coding regions (introns) of actively transcribed genes or intergenic regions [28]. Thus, we were able to demonstrate the safety, long-term engraftment and sustained expression of our anti-viral constructs, but there were still insufficient HIV-1-resistant blood cells in these patients to expect to see a clinical benefit.

**Table 1:** Integration site analysis of blood from UPN0306 during ATI.

Chromosome	Start	End	# of Sequence Reads	Gene	Relative Location
chr19	18,384,645	18,384,747	1,690,418	KIAA1683	intron
chr19	9,442,260	9,442,322	1,049,697	ZNF559-ZNF177	intron
chr21	15,383,973	15,384,065	211	ANKRD20A11P	intergenic (3’)
chr19	324,134	324,282	84	MIER2	intron
chr2	20,368,493	20,368,580	60	SDC1	intergenic (5’)
chr1	121,485,117	121,485,410	56	EMBP1	intergenic (5’)
chr19	323,958	324,045	48	MIER2	intron
chr19	324,378	324,465	43	MIER2	intron
chr19	18,384,488	18,384,578	34	KIAA1683	intron
chr19	18,384,347	18,384,404	21	KIAA1683	intron

### 2.2. Second Generation Anti-HIV-1 Constructs

#### 2.2.1. Improved RNA Expression

In our previous trials we used a lentiviral vector that expressed high levels of three anti-HIV-1 RNAs from strong, independent RNA polymerase III promoters (U6 and VA1) [27]. It is possible that one reason for the low level of gene marking is the toxicity and loss of HSPC or their progeny due to high levels of expression of RNA from the U6 promoters [29]. In order to optimize small RNA expression and minimize the potential toxicity from the over-expression of small RNAs, we evaluated RNA expression from a U1 promoter in the context of a naturally occurring micro-RNA cluster platform (MCM7), which ensures multiplexing and reduces the level of expression of the small RNAs. Northern blot analysis demonstrated a significant reduction in the level of the shRNAs expressed from the U1 promoter (compared to the U6 promoter), although we still observed potent inhibition of HIV-1 infection using a standard *in vitro* HIV-1 infectivity assay [30]. Among the constructs showing the greatest efficacy was one that contained combinations of 3 short hairpin RNAs (shRNAs) targeting HIV-1 *tat* and *rev* sequences.

#### 2.2.2. Selective Enrichment of Gene-Modified Cells

In order to further enhance the level of genetically-modified (HIV-1-resistant) peripheral blood cells in these patients, we propose that selective enrichment of gene-modified cells via drug selection is a more practical approach than relying on viral selective pressure during ATI. A cellular protein, methyl-guanine methyl-transferase (MGMT), repairs alkylating agent damage in cells, but is sensitive to the inhibitory effects of O^6^ benzylguanine (O^6^BG). Thus, treatment of cells with O^6^BG and BCNU results in the death of the cells. The P140 mutant of MGMT (MGMT^P140^) is resistant to inhibition with O^6^ benzylguanine [31] and thus, cells expressing MGMT^P140^ will survive O^6^BG and BCNU treatment [32,33,34,35,36]. Perhaps the most compelling evidence for the efficacy of this approach was demonstrated in a recent study by Younan and colleagues [37], who transplanted pigtail macaques with autologous HSPC modified to express an HIV-1 entry inhibitor (mC46) plus MGMT^p140^. Animals were conditioned with total body irradiation and the progeny of GM-HSPC were enriched to approximately 20% of all white blood cells with O^6^BG and BCNU treatment. After recovery, animals were infected with simian HIV (SHIV) and followed for SHIV viral loads, CD4 T-cell count and immune function. An animal that received mC46/MGMT^p140^-modified HSPC demonstrated selective survival of gene-modified CD4+ T cells in the blood, gastrointestinal tract and lymph nodes, reaching >90% of all CD4+ T cells at one point. A monkey receiving identical treatments, but whose HSPC were transduced with a (negative) control vector, had progressive SHIV infection and loss of CD4+ T cells. A two-to-three log reduction of SHIV viral load was observed in the mC46/MGMT^p140^-expressing animal, and the frequency of T-cell responses to HIV-1 Gag and Pol peptides was dramatically enhanced *vs*. the control animal. A similar increase in humoral responses to SHIV antigens was also observed. Importantly, the presence of gene-modified T cells afforded protection to the non-modified T cells in the same animal and resulted in a substantial recovery of immune function, including anti-HIV-1 responses in both the protected (gene modified) and unprotected CD4+ cell populations. These results indicate that protection of only a fraction (~20%) of T cells from HIV-1 infection may be sufficient to maintain an anti-HIV-1 immune response, control viremia and maintain CD4+ T-cell counts. Thus, transfer of autologous GM-HSPC has the potential to produce an HIV-1-resistant immune system that can prevent disease progression, even in cases where significantly fewer than 100% of the blood cells are protected. In addition, the use of the MGMT^p140^ was demonstrated to facilitate *in vivo* drug selection and enrichment of gene modified cells. We have thus included MGMT^p140^ gene in our second generation lentiviral vectors and are currently testing the ability to enrich for GM-HSPC (and their progeny) *in vivo* using a “humanized” mouse transplant model.

### 2.3. Patient Selection

HSPC transplantation requires concomitant cytotoxic therapy to permit engraftment of new stem cells. This is associated with toxicity, depending on the dose intensity of the cytotoxic therapy. AIDS patients having a therapeutic need for such therapy for other reasons are ideal candidates for early phase studies of this type, and it is for this reason that the first use of lentivirus stem cell therapy was performed in patients receiving high dose chemotherapy and autologous stem cell transplantation for AIDS-related lymphoma [26]. Even if the safety and feasibility of transplanting GM-HSPC in HIV-1 lymphoma patients can be established and we can develop safe methods that address the major issues of prolonged gene expression, level of engraftment, and selection of the genetically-modified progeny cells, the question is: how can this high-risk approach be moved into the non-malignant HIV-1/AIDS population? For the field to progress, one must design a study in HIV-1/AIDS that observes the appropriate risk: benefit requirements and give considerable consideration to such analysis for the population under study. Such risk: benefit analyses are strongly supported by the Food and Drug Administration (FDA), which provides Guidance that encourages use of reasonable assumptions and the development of risk-management plans [38]. In addition, the goal must be a therapy with reasonable likelihood that if successful, could become available to the group which endured the risks of testing. Thus, for example, the use of GM-HSPC in impoverished countries is so unlikely at this time, that including patients in such areas would not be appropriate for this study design. In today’s world, with rapidly increasing cost of medical care, this is as important as the risk considerations.

#### 2.3.1. Risk:Benefit Considerations

The analysis of the appropriate target population always involves the view of the sponsor, the treating physician, and the patient [39] (see also the FDA-sponsored seminar on the Patient Perspective in HIV-1 Cure Research [40]). For most HSPC-based gene therapies currently being tested and developed, the ultimate goal is to offer at least control of HIV-1 infection without concurrent cART, a so-called “functional” cure. It is even possible that this treatment will eradicate the HIV-1 reservoir and result in a “sterilizing” cure of the HIV-1 infection. However, early development and testing of such gene therapies is normally initiated in the patient group where the balance between risk and benefit does not place patients in a situation where foreseeable risks are higher than benefit. In later stages of therapeutic development, when safety of HSPC-based gene therapy approaches has been confirmed in the early Phase I studies, the target population can be changed so that efficacy can be tested for an approvable indication.

As the relative risks and benefits are weighed, one can assume that (a) all HIV-1 infected patients would optimally be managed per current guidelines; and (b) the research treatment would not alter standard of care procedure [41]. Thus, newly diagnosed patients would not be eligible, and only those patients on cART and those having some intolerance related to that treatment would be eligible for early stage GM-HSPC trials. As shown in Table 2, the disease spectrum of HIV-1/AIDS can thus be roughly broken into 6 patient groups, including: (1) healthy non-viremic patients on cART; (2) asymptomatic patients who stop cART because of intolerable side-effects or “treatment fatigue”; (3) the non-viremic patients on cART who have an incomplete immune recovery of CD4 count to protect them from progressive AIDS-related syndromes; (4) patients who fail to control the HIV-1 levels on cART; (5) the patients with treated lymphoma or another cancer who are in remission; and (6) the patients with lymphoma or another cancer requiring salvage therapy, including chemotherapy plus autologous stem cell transplantation. In Group 1, the healthy AIDS patients who control HIV-1 viremia while on cART are unlikely to be an initial target of HSPC transplantation, as the risks associated with the conditioning procedure are higher, as currently understood, than the benefit of continuing cART. In contrast, Group 2 patients, who cannot tolerate current cART, could benefit from use of an alternative therapy, and thus could be eligible for GM-HSPC strategies. However, some could argue that this group also needs an optimized cART-based treatment alternative before being exposed to the risks associated with conditioning and transplantation. The Group 3 non-viremic patients who are limited by an incomplete recovery of the CD4 count on cART and have limited therapeutic options is an obvious population for new treatment. These patients have a CD4 count <500 cells/µL, and ideally an HIV-1/AIDS patient should achieve a count of 500 cells/μL since this represents a level of immune restoration with morbidity and mortality similar to HIV-1-negative individuals [42]. In this group, despite control of HIV-1 plasma levels, patients are at risk for poor outcome [43,44,45]. This patient group varies based on the duration of cART treatment, CD4 count at onset of cART treatment, presence of inflammatory markers, and other factors [44]. Nevertheless, there is no specific approved treatment for any of these patients, and attempts to replace some of the antiretroviral medications in the cART regimen or treat them with IL2 and other immune enhancing agents have proven mostly unsuccessful (reviewed in [44]). Thus, based on ethical considerations, this population of HIV-1 infected patients balances the risks and benefits associated with a reduced-intensity conditioning regimen and GM-HSPC transplantation. Group 4, patients who have failed cART, would seemingly qualify for GM-HSPC clinical trials. However, as shown by studies that have closed during attempts to target this group, recruitment of these patients has become an increasingly difficult task, as cART improves. In addition, this group may have advanced disease, which limits the ability to mobilize HSPC and would create difficulties in generating a product sufficient for successful immune reconstitution [46].

Finally, the targeting of patients with recurrent or refractory lymphoma or other cancers (Group 6) has already been shown to be ideal for early gene therapy studies, since for these patients, ablative chemotherapy may already be indicated. However, the baseline risk for serious adverse events, such as secondary malignancy and myelodysplastic syndrome, estimated to be 10%–15% [47] could obscure the effects of the research procedure. In addition, the added conditioning therapy could further enhance this risk. These patients are also present in small number, and their robust recruitment to clinical trials is difficult. Most importantly, targeting this group begs the question of how will the derived data be relevant to the eventual application to the HIV-1/AIDS patient. Thus, although recruitment of patients from Group 6 can be justified in early phase GM-HSPC trials, such study design is limited.

### 2.4. Stem Cell Number and Quality

#### 2.4.1. HSPC Collection

The harvest and *in vitro* manipulation of HSPC from HIV-1+ patients must take into consideration that HIV-1 infection, cART treatment duration and co-morbidities are likely to have an effect on the number and quality of HSPC in these patients [48,49,50,51]. It is well established that HSPC from HIV-1+ patients have deficiencies in erythropoiesis, myelopoiesis and lymphopoiesis, but it is not clear which defects originate in the marrow stroma, HSPC or both [50,52,53,54]. Other studies do suggest that HIV-1 patients engraft stem cells at the same rate as HIV-1 negative transplant patients [55,56]. As described above, patients in our prior trial received high dose (marrow ablative) chemotherapy for their lymphoma prior to stem cell transplant [26]. In this first-in-human experience, safety considerations dictated that a fully engrafting dose of unmanipulated “backup” HSPC (>2 × 10^6^ CD34+ HSPC/kg) be infused immediately following the infusion of the gene-modified cells. With increased demonstration that lentivirus-transduced HSPC engraft well, current studies may require a “backup product,” that would only be infused only if patients fail to recover hematologically from the conditioning therapy. Thus, at present, it is recommended to collect ≥7.5 × 10^6^ CD34+ HSPC/kg from each patient to ensure that there are sufficient cells for producing both experimental and backup transplant products. This could be a problem depending on the group of HIV-1/AIDS patient targeted; for example, it has been shown that G-CSF mobilization can be limited in some AIDS patients [57]. Thus, we have elected to include plerixafor (AMD3100) in our stem cell mobilization protocols to enhance stem cell harvests from patients with lymphoma or multiple myeloma who fail to mobilize sufficient HSPC with standard G-CSF mobilization protocols [58].

**Table 2:** Framework for risk-benefit analysis for HIV-1-infected patient populations that could be targeted with HSPC-based gene therapy.

No.	HIV/AIDS Subpopulation	Current Rx Options for HIV-1 infection	Aspects of SOC Rx for HIV-1 infection	Potential Benefit of Research Rx	Real or Potential Risks of Research Rx	Risk:Benefit Analysis
1	HIV/AIDS pts on cART (controlled viremia and CD4 counts >500/µL)	cART	<10% treatment failure Outcome expectations excellent	Minimal to no potential benefit since virus control and CD4 counts are adequate	Transient myeloid dysfunction Unknown effects of genetic modification & HSPC mobilization	Unfavorable; first in human trial cannot be justified in this group
2	AIDS pts off cART (side effects to cART or cART “fatigue”)	Symptomatic Rx if cART not tolerable	Heightened potential for AIDS progression	Improved control of HIV-1	Transient myeloid dysfunction Unknown effects of genetic modification & HSPC mobilization	Favorable but conditioning adds unnecessary risk in these patients who are already drug adverse
3	AIDS pts on cART, with incomplete immune recovery with suboptimal CD4 levels	cART Treatment as indicated for infections	Poor expected outcome	Expansion of CD4 count Potential for improved control of HIV-1	Transient myeloid dysfunction Unknown effects of genetic modification & HSPC mobilization	Favorable
4	AIDS pts who do not respond to cART	Research therapy with new antivirals	Poor expected outcome	Improved control of HIV-1	Transient myeloid dysfunction Unknown effects of genetic modification & HSPC mobilization	Favorable but limitation of subject availability
5	ARL pts in remission following frontline Rx	cART Treatment as indicated for infections	Remission stable Outcome expectations very good; concern for risk of myelodysplasia	Minimal to no potential benefit IF virus control and CD4 counts are adequate	Transient myeloid dysfunction Unknown effects of genetic modification	Less favorable due to potential for myelo-dysplasia post-chemotherapy and conditioning
6	ARL pts on salvage therapy (transplant)	cART Treatment as indicated for infections	Outcome expectations good; concern for myelodysplasia risk	Minimal to no potential benefit IF virus control and CD4 counts are adequate	Transient myeloid dysfunction Unknown effects of genetic modification	Less favorable due to 10%–20% potential for myelodysplasia post-transplant and conditioning

Abbreviations: Rx = treatment; cART = combination antiretroviral therapy; SOC—standard of care; ARL = AIDS-related lymphoma.

#### 2.4.2. HSPC and HIV-1 Infection

In addition to potential limitations in the hematopoietic potential of the HSPC from patients with long-standing HIV-1 infection, several groups have now reported that HSPC can be infected with HIV-1 and may in fact harbor a latent reservoir of virus [59,60,61] although other evidence suggests that this may not be the case [62]. In one study, 6 of 11 patients on long-term cART had evidence of integrated provirus in HSPC even after 8 years of no detectable virus in blood samples [63]. The presence of proviral HIV-1 sequences in HSPC was more pronounced in patients who were more recently diagnosed, but who remained below the limit of detection of virus in the blood. If true, this raises significant concerns for the use of autologous stem cells for transplantation of HIV-1+ patients. However, the hematopoietic potential and extent of latent virus in more primitive HSPC in individuals on long-term cART is unclear. Since the success of the transduction, engraftment and lineage development is critically dependent on the hematopoietic potential of these cells, further investigation is warranted. Finally, it is not known if gene modified (HIV-1 resistant) T cells will have normal immune function or will exhibit an “exhausted” phenotype as is seen in chronically infected patients on cART [54,64]. A similar case can be made for the function of CD4+ monocytes and dendritic cells, as all three cell types may also harbor latent virus. Thus, additional work is needed to ensure the biological function of HIV-1-resistant blood cell populations. 

### 2.5. Preparation for Transplantation

Current clinical reports for HSPC-based gene therapy demonstrate that patients require either myeloablative or reduced intensity (non-ablative) conditioning of the bone marrow to create “space” for the GM-HSPC to engraft. Risk management of this complication is based on close monitoring for fever and early use of antibiotics. However, the long-term risk of malignancy associated with any chemotherapy needs to be weighed carefully in healthy HIV-1 patients. Regarding the potential for genotoxicity associated with gene-therapy, risk has to be evaluated for each specific case and taken into account in the risk:benefit analysis. Furthermore, risks associated with stem cell mobilization, apheresis and transplantation weigh differently in each of the patient groups analyzed, depending on the therapeutic needs, existing condition, and accumulated risks. Severe toxicity is possible for patients concurrently receiving ritonavir-based (boosted-PI) therapy and the immunosuppressive agents, antifungals and high dose chemotherapy. Patients receiving ritonavir throughout conditioning are managed differently than those who are not. This is due to the potent inhibition of CYP3A4 and P-glycoprotein by ritonavir, resulting in the delayed metabolism and consequently higher serum levels of certain chemotherapeutic agents and antifungals. This is of particular importance in the setting of high-dose, ablative therapy, where the therapeutic/toxic window is narrow.

Two recent reports on HSPC gene therapy for metachromatic leukodystrophy [65] and Wiskott-Aldrich syndrome [66] employed myeloablative conditioning and reduced intensity myeloablative conditioning, respectively, prior to infusion of GM-HSPC. Patients in the leukodystrophy trial received a targeted 4200–5600 µg/L*hour (AUC adjusted) busulfan from day −4 to day −1 prior to transplantation. Although the conditioning regimen was well tolerated, patients experienced neutropenia (absolute neutrophil count < 500/µL) from as early as day +9 up to +45 after transplantation. Reduced-intensity conditioning with chemotherapy is also expected to have transient side effects [67], and there is likely to be a period of neutropenia during which the risk of infection is increased. In the Wiskott-Aldrich trial, patients received a reduced intensity conditioning regimen of 7.6–10.1 mg/kg intravenous busulfan plus 60 mg/m^2^ of fludarabine and monoclonal antibody to CD20 (Rituximab^TM^). These patients also experienced neutropenia (ANC < 500/µL) lasting from 12–19 days, but ultimately recovered without the use of backup HSPC products. It is thus questionable whether the risk of using these levels of busulfan is acceptable for HIV-1/AIDS patients who have a significantly better prognosis than the patients in these trials.

Non-myeloablative busulfan-based conditioning regimens have been used prior to hematopoietic cell transplant in chronic myelogenous leukemia [68,69], but have more recently been used for autologous transplant and GM-HSPC transfer approaches in non-malignant diseases, including immunodeficiencies (T and B cell), β-thalassemia, X-linked adrenoleukodystrophy, and chronic granulomatous disease [67,70,71,72,73]. Results of a recent clinical study of gene therapy for adenosine-deaminase-deficient severe combined immune deficiency (ADA-SCID) demonstrated that non-myeloablative pre-transplant conditioning with low-dose busulfan (65–90 mg/m^2^) had a positive impact on achieving therapeutic benefits with gene-therapy for ADA-SCID [67]. The 10 patients enrolled in the study were between 15 months and 20 years old. The study directly compared two different approaches to gene therapy for ADA-SCID: the first cohort of 4 patients did not receive cytoreductive conditioning before infusion of transduced CD34+ cells and continued to have ADA-enzyme replacement therapy (ERT), whereas the following 6 patients had non-myeloablative conditioning with busulfan and had ERT withdrawn. Long-term presence of gene-containing blood cells expressing ADA activity was demonstrated only in the cohort with busulfan conditioning and withdrawal of ERT. The authors argued that conditioning with busulfan was responsible for this success, although it is difficult to quantify the selective impact of ERT withdrawal. All patients who received busulfan experienced transient neutropenia (without any adverse consequences), thrombocytopenia, and elevation of liver enzymes. After 3–5 years, 3 patients were well without ERT.

Two other studies provide supporting data on the use of low-dose busulfan for conditioning prior to gene-therapy product infusion [70,71]. Children (0.6 to 5.6 year old) received CD34+ cells transduced with an ADA-containing retroviral vector after a non-myeloablative conditioning with 2 mg/kg/day on days 3 and 2 before gene therapy [70]. After a median duration of follow-up of 4 years, busulfan conditioning and gene therapy were not associated with serious adverse events. Nine of the 10 patients had the immune function restored and were protected against severe infection. The authors concluded that use of the non-myeloablative regimen and withdrawal of PEG-ADA (to allow for natural selection of gene corrected cells) was a crucial factor in the success of the trial. Conditioning with busulfan was also used at a total dose of 8 mg/kg (liposomal busulfan, intravenous) in a GM-HSPC trial of X-linked chronic granulomatous disease [74]. Gene marking in peripheral blood leukocytes was detected at sustained levels between 10% and 30% for the first 3–4 months after transplantation, without busulfan-related significant adverse events other than a Grade 1 mucositis episode. Thus, despite some variability in the busulfan dosing strategies, all of which were below the ablative threshold of 9 mg/kg, numerous examples of the efficacy of non-myeloablative conditioning in promoting engraftment of GM-HSPC in non-malignant patients supports the concept of its use in the HIV-1/AIDS patient population.

### 2.6. Getting to Market

In order for a stem cell based therapy to make its way to standard of care for HIV-1/AIDS, it must demonstrate two fundamental properties. First, the cell therapy must demonstrate a significant improvement in slowing or preventing disease progression (relative to standard of care treatment) or provide a cure (functional or sterilizing). Second, the therapy must reduce the overall cost of treatment to be economically viable. To address the first point, it is logical to propose the use of surrogate endpoints, such as control of viremia and recovery of CD4 counts as surrogate study endpoints for licensure trials. The use of surrogate endpoints is critical for the development of HIV-1 therapeutics since (a) unlike cancer, the course of disease in AIDS has been prolonged by the use of antiretroviral drugs; and (b) traditional endpoints, such as overall survival and disease free survival, are not obtainable in a reasonable timeframe for drug development. Instead, as outlined in 210 CFR 601 Subpart E (Accelerated Approval of Biological Products for Serious or Life-Threatening Illnesses) “*FDA may grant marketing approval for a biological product on the basis of adequate and well-controlled clinical trials establishing that the biological product has an effect on a surrogate endpoint that is reasonably likely, based on epidemiologic, therapeutic, pathophysiologic, or other evidence, to predict clinical benefit or on the basis of an effect on a clinical endpoint other than survival or irreversible morbidity.*” Drug approval under these conditions is likely to play a major role in the development of a first generation HIV-1 gene therapy product, as it satisfies concerns for investors on time to market and creates a pathway for cell based therapies that accepts that cell products are unique and should not be regulated like drugs for cancer or other acute life threatening diseases. Thus, in addition to following the level of gene marking in the blood, lymph nodes and rectal mucosa before and after drug selection, our proof of concept studies will include surrogate endpoint measurements, such as viral load and CD4 T-cell counts, as likely indicators of efficacy. In addition, we will use ATI as a clinical indicator that cell-based control of virus and rebound of CD4 T-cell counts result in the immunological clearance of latent viral reservoirs.

The economic viability of stem cell gene therapy has been called into question and has been shunned by many as not a plausible alternative to drug therapy [75], but realistic estimates of the potential economic impact of successful gene therapy for HIV-1 suggest that this type of treatment could be cost effective [76]. For example, the cost of an autologous bone marrow transplant ranges from $40,000 to upwards of $90,000, depending on patient status and complications following infusion [77]. When adding the costs for genetic modification and required product release testing, we estimate a cost of goods (COG) ranging from $120,000–$150,000. It is reasonable to assume a 100% mark-up in costs to deliver the product to market, resulting in a final cost of $250,000–$300,000/patient. By comparison, the cost of lifelong cART therapy has been estimated to be as high as €320,000–€575,000 ($420,000–$755,000) with 73% of the cost going towards cART drugs [78]. The conversion to generic first line combination cART drugs (1-pill efavirenz-emtricitabine-tenofovir) is estimated to only reduce costs about $42,000 per patient [79]. Thus, the proposition that a one-time intervention with stem cell gene therapy is not outside of economic reality in developed countries. Success in these areas may lead to COG savings related to volume, and thus the therapy may also be able to reach affordability in more economically challenged areas.

## 3. Experimental Section

*Analysis of vector marking and gene expression.* DNA and RNA were isolated from the peripheral blood of patients at the indicated time points and analyzed for gene marking and RNA expression as previously described [26].

*Analytical Treatment interruption.* One subject (UPN0306) was aviremic by standard of care assay (<50 gc/mL) with CD4 counts > 500 cells/µL and had detectable lentiviral vector sequences cells (by WPRE PCR assay) within the peripheral blood and underwent an ATI after informed consent according to City of Hope Institutional Review Board approved protocols. The anti-retroviral treatment was discontinued and the subject was examined every other week with collection of laboratory samples during the entire treatment interruption. cART was resumed at 21 days after initiation of ATI following viremia >100,000 HIV copies/mL and CD4 counts < 200 cells/µL.

*Integration site analysis.* Peripheral blood was collected from UPN0306 at 5 weeks after the initiation of ATI and DNA was extracted using a QIAamp DNA Blood Mini Kit (Qiagen, Valencia, CA, USA). Analysis of integration site was performed as previously described [80]. Briefly, single strand DNA amplicons were produced from genomic DNA from a WPRE-specific primer by ssPCR. A random ssDNA primer was then ligated to the 3’ end of the extended DNA and 100 cycles of nested PCR was run to produce template for sequencing analysis. Sequence analysis was performed using HiSeq methods as previously described [81]. The sequences generated from Illumina’s base calling pipeline that passed default filters were kept for further analysis. Sequences were then aligned to hg19 genome with Bowtie v0.12.8 using default settings. Further analysis was done using R and Bioconductor packages. The regions that were covered by at least 10 reads were identified and annotated to refseq genes downloaded on 11 September 2012 from UCSC genome database with “ChIPpeakAnno” package. 

## 4. Conclusions

In summary, several early stage clinical trials have demonstrated the safety and feasibility of stem cell gene therapy for HIV-1/AIDS, but none have resulted in objective improvement of disease state. The data presented suggests that pre-conditioning of the marrow space is required for long term engraftment of gene modified cells, but *in vivo* enrichment of these cells may be needed to reach therapeutic levels of engraftment. We have developed a new generation of viral vectors and a clinical strategy to test this hypothesis and move this treatment into proof of concept studies and commercial product development. This strategy is intended to complement current treatment modalities (drugs, vaccines) and, where appropriate, to replace current therapy altogether with single treatment cell therapy resulting in a functional or sterilizing cure to HIV-1/AIDS.
